# Nerve Diseases

**Published:** 1905-09-02

**Authors:** 


### NERVE DISEASES.

**Cortical Localisation.**—During the past few years considerable advances have been made in our knowledge of the extent of the motor cortex in the anthropoid apes, which have an immediate practical bearing on the application of surgical methods to the treatment of tumours of the brain. Up to the end of the nineteenth century only monkeys and one orang had been investigated. In monkeys it had been found that the motor cortex lay on both sides of the fissure of Rolando, in the ascending frontal and ascending parietal convolutions, and that the areas or centres for various movements lay very close together. In the orang the same distribution of the motor area was found, the chief difference from monkeys being that the various centres from which movements could be excited by electrical stimulation were separated from one another by spaces in which stimulation called forth no response. In more recent years the chimpanzee and gorilla have been the subjects of experiments, by Sherrington and Grünbaum. In these animals, especially in the latter, the configuration of the various gyri and sulci of the cerebral cortex is much closer to what obtains in man than is the case in the orang and lower monkeys. It is probable, therefore, that the distribution of the motor area in them is more nearly what it is in man. In the chimpanzee and gorilla it was found that the motor area is entirely in front of the fissure of Rolando; excitation of the ascending parietal convolution calling forth no motor response. The excitable area is confined to the ascending frontal convolution, and the cortex immediately in front of this. The order of the centres, from above downwards, is leg, trunk, arm, neck, and face. These centres are separated by inexcitable intervals just as in the orang. These results, obtained by electrical stimulation, were confirmed by ablative experiments. Excision of areas in the ascending frontal convolution was followed by paralysis and degeneration of the pyramidal tract; whereas excision of areas in the ascending parietal convolution had no such results. More recently these experiments of Sherrington and Grünbaum have been confirmed and extended to the human cortex by Campbell. He found that the giant cells of Betz—which give rise to the pyramidal fibres—are confined to the motor cortex in the gorilla, none being found behind the floor of the fissure of Rolando. In man, too, these cells are not present behind the fissure of Rolando. It seems almost certain, therefore, that the motor area in man is altogether in front of that fissure.[1]

**Myasthenia gravis.**—The disease called myasthenia gravis, which was, until a few years ago, confounded with bulbar palsy, has gradually been isolated and its pathology elucidated, though as yet no satisfactory treatment has been found.[2] The patient generally complains of muscular weakness, which is very apt to be marked in the levatores palpebrarum, causing ptosis, and there may be squint as well. The great characteristic of the complaint is that the patient begins a movement well and easily but very soon tires, and, for instance, when beginning to speak articulation is clear and the voice good; as he proceeds the tone becomes lower and nasal in character, and he finally becomes both voiceless and breathless. There is a similar reaction to faradic electrical stimulation; at first the response of the muscle is good, it gets more feeble, and in time disappears altogether. Post-mortem examination of the central and peripheral nervous system and of the muscles reveals no change whatever; so that it has been supposed that the disease is due to some toxin which affects the terminal nervous structures in the muscles. In some cases definite enlargement of the thymus or multiple lymphoid tumours scattered in the muscles have been found, and possibly the toxin is formed by these abnormal structures. The diagnosis is often difficult, and the earlier stages may easily be confounded with hysteria, bulbar palsy, or post-diphtheritic palsy. The distinctive characters mentioned above will, however, generally enable a diagnosis to be made. Some patients will go on for many years, but in others the disease rapidly progresses, causing death from respiratory paralysis.

[1] Scottish Med. Jour., May. [2] Brit. Med. Jour., March 11.

